# Report of the Convalescent Homes Committee

**Published:** 1920-12-25

**Authors:** 


### REPORT OF THE CONVALESCENT HOMES COMMITTEE.

1. The President and General Council have this year fixed the sum available for distribution amongst convalescent homes and consumption sanatoria at £10,000, as against £12,000 in 1919.

2. The number of applications eligible for consideration amounted to fifty-one from convalescent homes and eleven from consumption sanatoria, as against forty-nine and nine respectively last year.

3. In view of the decreased amount available for distribution the Committee have taken into consideration, even more than usual, the question of comparative urgency of need, as shown by the audited accounts and balance sheets of the various institutions to December 31 last. In the case of consumption sanatoria they have also taken note of the proportion of the total work of each institution which is covered by the receipts from public authorities. As these and other circumstances affecting the relative claims of the different institutions vary from year to year, it must not be assumed that a reduction in the grant to any particular institution, whether for maintenance or for capital purposes, or even the absence of a grant, implies dissatisfaction.

4. The convalescent homes which were reopened after the war during the course of 1919 have now been doing their normal work for a whole year, and the amount allotted to convalescent homes has therefore had to be further increased.

5. This fact, combined with the diminution in the total placed at the disposal of the Committee, has reduced the amount available for distribution to consumption sanatoria in consideration of the reservation of beds for the use of patients in London hospitals, and as the cost of maintaining beds has continued to increase, the Committee have been reluctantly compelled to make a substantial reduction in the number of beds so reserved. The accommodation secured this year amounts to forty-four beds at six sanatoria, situated as follows: Ten beds for children at the Children's Sanatorium, Holt, Norfolk; two beds at the Daneswood Sanatorium (Jewish), Woburn Sands, Beds; eight beds at the Devon and Cornwall Sanatorium, South Brent, Devon; four beds at the Kelling Sanatorium, Holt, Norfolk; twelve beds at the Sanatorium of the National Association for the Establishment and Maintenance of Sanatoria for Workers, Benenden, Kent; eight beds at the Northamptonshire Sanatorium, Creaton, Northants.

6. The Committee recommend that the beds thus reserved be allocated amongst London hospitals as follows: Chest hospitals: Eight beds to the City of London Hospital for Diseases of the Chest; four beds to the Royal Chest Hospital. General hospitals: Two beds to Charing Cross Hospital; five beds to Guy's Hospital; two beds to King's College Hospital; seven beds to the London Hospital; three beds to the Middlesex Hospital; two beds to the Royal Free Hospital; two beds to St. George's Hospital; one bed to St. Mary's Hospital; five beds to St. Thomas's Hospital; two beds to University College Hospital; one bed to Westminster Hospital.

7. In view of the necessity of reducing the number of beds, the Committee held a conference of representatives of the hospitals concerned in order to discuss the best method of using the beds that would be available. There was general agreement that, while most of the patients were theoretically eligible for treatment by the public authorities, it was a great advantage to the hospitals to have immediate access for suitable cases to the beds reserved by the Fund. Although the Committee recognise that a time may come when the funds at their disposal can be more usefully applied in some other direction, they have no hesitation in recommending that the reservation of beds should be continued for the present.

8. The Committee are greatly indebted to the visitors who so kindly volunteered for the inspection of consumption sanatoria, often at a considerable distance from London.

9. The grants recommended to the various institutions are appended.

For the Committee, William H. Bennett, Chairman.

November 25, 1920.

(*Continued on page* 302.)

King Edward's Hospital Fund for London—(continued from p. 290).

### Grants Recommended by the Convalescent Homes Committee.

*The Convalescent Homes Committee desire to draw attention to the fact that it must not be assumed that the reduction or absence of a grant implies dissatisfaction.*

A.—CONSUMPTION SANATORIA.

Children's Sanatorium, Holt, Norfolk, £880, of which £780 in consideration of the reservation of ten children's beds during 1921 for the use of certain London hospitals (see paragraphs 5 and 6 of Report). Daneswood Sanatorium (Jewish), Woburn Sands, £270, in consideration of the reservation of two beds during 1921 for the use of certain London hospitals (see paragraphs 5 and 6 of Report). Devon and Cornwall Sanatorium, Didworthy, South Brent, £1,130, of which £1,080 in consideration of the reservation of eight beds during 1921 for the use of certain London hospitals (see paragraphs 5 and 6 of Report). Eversfield Chest Hospital, St. Leonards, £50. Fairlight Sanatorium, Hastings, £100. Firs Home, Bournemouth, —. Kelling Sanatorium, Holt, Norfolk, £690, of which £50 to new disinfector, and £540 in consideration of the reservation of four beds during 1921 for the use of certain London hospitals (see paragraphs 5 and 6 of Report). Mount Vernon Hospital, Northwood, £100. National Association for the Establishment and Maintenance of Sanatoria for Workers, Benenden, Kent, £1,870, of which £1,620 in consideration of the reservation of twelve beds during 1921 for the use of certain London hospitals (see paragraphs 5 and 6 of Report). Northamptonshire Sanatorium, Creaton, Northants, £1,130, of which £1,080 in consideration of the reservation of eight beds during 1921 for the use of certain London hospitals (see paragraphs 5 and 6 of Report). Royal National Hospital for Consumption, Ventnor, £200. Total, £6,420.

B.—CONVALESCENT HOMES.

All Saints' Convalescent Home, St. Leonards, £25. Beau Site Convalescent Home, Hastings, £50. Brentwood Convalescent Home for London Children, Brentwood, £50. Brooklands Home (Invalid Children's Aid Association), Worthing, £25. Bushey and Bushey Heath Children's Convalescent Home, Bushey, £50. Chelsea Hospital for Women, St. Leonards, £75, with a view to encouraging the admission of patients direct from the hospital. Children's Convalescent Home, Beaconsfield, £25. Children's Cottage Hospital, Cold Ash, Newbury, £25, in consideration of convalescent cases. Children's Home Hospital, Barnet, £25, in consideration of convalescent cases. Clevedon Sanatorium (Invalid Children's Aid Association), Broadstairs, £25. Convalescent Home for Poor Children, St. Leonards, £150. Convalescent Police Seaside Home, Hove, £80. E. G. Anderson Hospital (Home of Recovery), Barnet, £250, of which £50 to reduce debt. East London Hospital for Children, Bognor, £50. Florence Emma Home (Invalid Children's Aid Association), Dover, £25. Friendly Societies' Convalescent Homes, Dover and Herne Bay, £100. Great Northern Central Hospital, Clacton, £125. Great Northern Central Hospital, East Finchley, £150. Highgate Convalescent Home for Children, Highgate, £50. Jewish Convalescent Home (Children), Brighton, £75. King's College Hospital, Hemel Hempstead, £150 (the grant is made on condition that patients are admitted direct from the hospital). London and Brighton Female Convalescent Home, Brighton, £25. Metropolitan Convalescent Institution, Bexhill, £50. Metropolitan Convalescent Institution (Children), Broadstairs, £100. Metropolitan Convalescent Institution, Walton, £150. Middlesex Hospital, Clacton-on-Sea, £250. Miller Hospital Convalescent Home, Bexhill, £100. National Hospital for the Paralysed and Epileptic, East Finchley, £125. Paddington Green Children's Hospital, Slough, £100. Poplar Hospital, Walton-on-Naze, £50. Queen Charlotte's Lying-in Hospital, Kilburn, £75. Queen's Hospital for Children, Bexhill, £100. Royal Waterloo Hospital for Children and Women, Whippingham, £75. St. Andrew's Convalescent Home, Folkestone, £100. St. Andrew's Convalescent Hospital, Clewer, £25. St. George's Hospital, Wimbledon, £100. St. Luke's Home (Children), Woodley, Reading, £25, in consideration of convalescent cases. St. Mary's Convalescent Home, Birchington, £25. St. Mary's Home for Children, Broadstairs, £75, in consideration of convalescent cases. St. Michael's, Westgate, £25. St. Peter's Holiday House, St. Leonards, £25. Samaritan Free Hospital, Amersham, £50. Seaside, Seaford, £50. Sunday School Union, Bournemouth, £25. Sunshine Home, Hurstpierpoint, £50. Surgical Home for Boys, Banstead, £25, in consideration of convalescent cases. Tilford Convalescent Home for Children, Farnham, £25. Victoria Home for Invalid Children, Margate, £25, in consideration of convalescent cases. Victoria Hospital for Children, Broadstairs, £150. Total, £3,580.

| | |
|---|---|
| Grants to Consumption Sanatoria ... ... ... ... | £6,420 |
| Grants to Convalescent Homes ... ... ... ... | 3,580 |
| Total ... ... ... ... ... | £10,000 |

